# Interventions maintaining eating Independence in nursing home residents: a multicentre qualitative study

**DOI:** 10.1186/s12877-018-0985-y

**Published:** 2018-11-27

**Authors:** Alvisa Palese, Valentina Bressan, Tea Kasa, Marin Meri, Mark Hayter, Roger Watson

**Affiliations:** 1 Department of Medical Sciences, University of Udine, Viale Ungheria, 20, 33100 Udine, Italy; 2 Faculty of Health Sciences, University of Hull, Cottingham Road, HU6 7RX Hull, UK

**Keywords:** Feeding difficulties, Mealtime difficulties, Eating assistance, interventions, Dementia, Elderly, Tacit knowledge, Perceived effectiveness, Nursing home, Qualitative study, Content analysis

## Abstract

**Background:**

Despite 32 years of research and 13 reviews published in the field, no intervention can be considered a gold standard for maintaining eating performance among residents with dementia. The study aim was to highlight the interventions derived from tacit knowledge and offered daily in assisting eating by healthcare professionals (HCPs) in nursing homes (NHs).

**Method:**

A multicentre descriptive qualitative study was performed in 2017. Thirteen NHs admitting residents with moderate/severe functional dependence in eating mainly due to dementia, were approached. A purposeful sample of 54 HCPs involved on a daily basis in assisting residents during mealtime were interviewed in 13 focus groups. Data analysis was conducted via qualitative content analysis.

**Results:**

The promotion and maintenance of eating performance for as long as possible is ensured by a set of interventions targeting three levels: (a) environmental, by ‘Ritualising the mealtime experience by creating a controlled stimulated environment’; (b) social, by ‘Structuring effective mealtime social interactions’; and (c) individual, by ‘Individualising eating care’ for each resident.

**Conclusions:**

In NHs, the eating decline is juxtaposed with complex interventions regulated on a daily basis and targeting the environment, the social interactions, and the residents’ needs. Several interventions that emerged as effective, according to the experience of participants, have never been documented before; while others are in contrast to the evidence documented. This suggests the need for further studies in the field; as no conclusions regarding the best interventions have been established to date.

## Background

Functional dependence has been documented as the primary reason for nursing home (NH) admissions among older people [1]. According to a hierarchical order already documented [2] eating dependence is one of the late-loss Activity of Daily Living (ADL) mainly associated to cognitive impairments or dementia [3]. Eating and drinking are both vital to providing energy, nutrients, and an adequate intake, as well as to affect physiological, psychological, and social well-being and quality of life [4]. However, in the NH context, eating dependence is one of the most complex needs to satisfy due to the high prevalence of residents with eating difficulties and their different degrees of dependence, their specific preferences and routines [5], which have all been reported as challenging the intent of healthcare professionals (HCPs) to maintain residents’ independence for as long as possible [6].

In the first stage of dementia, eating difficulties manifest usually as a refusal to eat as taste and smell change, and major depression can trigger anorexia and weight loss [7, 8]. Loss of appetite, behavioural disorders, and apraxia are typical symptoms in the moderate stages of dementia whereas in advanced stages, a decline in all ADLs, including eating, becomes evident. In the late stages of dementia, older people have been shown to be unable to recognise food, to use cutlery appropriately, to take or keep food in the mouth, chew, and swallow [8–10]. As a consequence, an increased likelihood of malnutrition, anorexia, pressure injuries, dehydration, aspiration pneumonia, multiple hospitalisations and mortality, have been documented [3, 11].

The effectiveness of interventions aimed at promoting and maintaining eating performance over time among older people with dementia has been explored in many studies, reviews and meta-analyses (e.g., Abdelhamid et al. [12], Bunn et al. [13], Abbott et al. [14]). However, despite 32 years of research started by the first study in the field published by Eaton et al. [15] and followed by several primary studies summarised in 13 reviews [2, 3, 9, 12–14, 16–22], no conclusive evidence-based intervention(s) have been established to date [13]. Substantially, what works better in maintaining eating independence as long as possible and postponing the use of feeding tubes [18] at the individual (e.g., handfeeding technique) and at the environmental levels (e.g., music to increase meal intake [23]) has not been established to date. As a consequence, any intervention can be recommended as of now as a gold standard [12].

In a field of research where evidence is still lacking, consulting expert practitioners and trying to discover their tacit knowledge as the knowledge-in-practice developed from direct experience and action, can represent a valuable opportunity for researchers. Furthermore, interviewing them with the aim of exploring their pragmatic and situation-specific knowledge, subconsciously understood and applied, shared through interactive conversation and experience [24], can develop new insights and expand future research. Therefore, this study explored NH professional tacit knowledge derived from professional experience and action of staff involved during mealtime in NHs. Specifically, the principal purpose of this study was to highlight interventions implemented on a daily basis according to their perceived effectiveness by HCPs aimed at maintaining eating independence among older people with dementia who live in NHs.

## Methods

### Study design

A descriptive qualitative study design [25] based upon focus group methodology [26] was undertaken in 2017 and reported here according to the COnsolidated criteria for REporting Qualitative research, which has been recommended as a checklist for explicit and comprehensive reporting of qualitative studies [27].

### Setting and participants

All public (9) and private (4) NHs regulated by the Regional Health Service in a rural area of a north-eastern Italian region were approached and agreed to participate. Residents admitted into these NHs with moderate to severe functional dependence were at need of assistance in eating [28] due to different clinical conditions, mainly dementia. According to the regional rules, at the time of the study, NHs were offering an average of 75 min of nursing care per day for each resident.

A purposeful sample [29] of HCPs in each NH was identified by the nurse manager (NM) and the principal investigator (AP) according to the following inclusion criteria: (a) those who were involved on a daily basis in assisting residents at mealtimes; (b) those with at least of six months of NH experience; and (c) those who were willing to participate in the study. All HCPs invited agreed to participate: in Table 1, the main NHs and focus group participant profiles are reported.

**Table 1:** Nursing Home Bed Size, Residents Hosted, and Focus Group Members Involved in the Study

NH	Bed size, *n*	Residents living in the NH, *n*	Focus group participants, *n*	Professional roles, *n*	F/M	Age, mean (*SD*) in years	Experience in the NH, mean (*SD*) in years
1	49	46	3	2 NAs, 1 PHYS	3/0	39.3 (10.0)	10.0 (8.0)
2	200	164	4	3 NAs, 1 RNs	4/0	49.2 (8.4)	18.5 (5.6)
3	32	30	3	2 NAs, 1 PHYS	2/1	42.0 (5.6)	9.0 (2.8)
4	154	152	7	4 RNs, 3 NAs	3/1	40.5 (11.2)	8.2 (8.5)
5	102	95	6	3 NAs, 2 RNs, 1 ED	6/1	50.1 (5.2)	22.8 (9.1)
6	33	25	3	2 NAs, 1 RN	3/0	53.0 (11.3)	22.5 (7.6)
7	87	80	4	2 RNs, 2 NAs	3/1	37.0 (16.7)	10.3 (3.4)
8	61	55	3	2 NAs, 1 RN	3/0	54.3 (0.5)	15.3 (10.2)
9	86	80	5	4 NAs, 1 RN	5/0	54.2 (20.3)	8.2 3.2)
10	115	115	4	3 RNs, 1 NA	3/1	51.8 (12.9)	16.6 (14.3)
11	58	58	5	3 NAs, 2 RNs	5/0	49.0 (16.4)	9.6 (1.2)
12	118	117	3	2 RNs, 1 NAs	3/0	26.0 (5.0)	3.5 (1.2)
13	66	63	4	3 NAs, 1 RN	4/1	43.2 (17.6)	8.5 (5.3)
Total	1161	1080	54	31 NAs, 20 RNs, 2 PHYS, 1 ED	48/6	45.1 (13.4)	12.5 (11.0)

*ED* Professional Educator, *F* Female, *M* Male, *n* number, *NH* Nursing Home, *NA* Nurse Assistant, *PHYS* Physiotherapists, *RN* Registered Nurse, *SD* Standard Deviation

### Data collection process

Data were collected in two steps on a day identified by the NM in each NH. In the first step, four trained researchers (AP, KT, AD, ML) observed a lunchtime in each NH from start to finish. Researchers were present in the dining room as a non-disturbing presence, without directly approaching the resident tables and without speaking with the carers. They also observed residents who ate in other environments (e.g., their bedroom). During the observation process, researchers collected notes regarding HCPs behaviour and interactions with residents. These notes were recorded in a diary kept confidential and were then used during the second step as examples for anchoring questions in the real practice (e.g., ‘I observed during lunch that you switched off the television…’) as reported in Table 2. A total of 26 h of observation was performed by each researcher, reaching a total of 104 h of observation during the entire project.

**Table 2:** Open-Ended Questions Guide for Conducting Each Focus Group

*First phase* Aims: collecting demographic and professional data Questions: Can you report your professional profile, age, education, and professional experience? *Second phase* Aims: stimulating the emersion of interventions perceived as effective throughout conversation Questions: By considering your experience here in this NH … please focus your attention on those interventions that you perform on a daily basis with the intent of maintaining eating independence of residents. 1) Can you describe what kind of intervention(s) works better in terms of effectiveness in maintaining eating independence in residents with dementia? Can you describe in detail what condition (e.g., context, time) this/these intervention(s) are most effective according to your daily practice? 2) Can you describe the degree of adaptation required of these interventions on a daily basis, considering the needs of each resident and the needs of the entire group of residents cared for by you and the team? 3) Can you describe the order, if any, in which this/these intervention(s) should be implemented to aim for maximising their effectiveness on eating performance? 4) Can you describe the roles of family caregivers in the process of feeding assistance with regard to its effectiveness according to your experience? 5) Can you describe if the above-mentioned interventions are standardised or flexible across (a) residents, (b) time, (c) day (lunch vs. dinner), and (d) healthcare workers? 6) Can you describe the entire process of meal service from the beginning to the end by highlighting what intervention(s) should be carefully considered due to its capability to make a difference in resident eating independence? *Third phase* Aims: stimulating further discussion on interventions witnessed by researchers during lunchtime observation Question(s) anchored on the data emerged from the observations performed by researchers during lunchtime and reported in a diary. For example: ‘During lunchtime, we observed that you offered a specific seat to a resident and left him alone for the entire meal … can you explain the reason(s) for this decision and its impact on eating independence?’ *Fourth phase* Aims: accommodating new insights and data into what has already been collected by discussing those interventions that emerged in the previous focus groups Question(s) anchored on the interventions that emerged in the previous focus groups. For example: ‘In the previous focus group(s), colleagues reported that television and music used to be avoided during mealtimes to limit distractions…What is your experience with regard to this intervention? Is this an effective intervention to maintain eating performance? *Focus group conclusion* Do you have further comments or other points to add?	

*NH* Nursing Home

The second step of the study was performed just after the observation in each NH. The HCPs observed were purposefully selected and asked to participated in the focus group. A total of 13 focus group interviews were conducted, one for each NH, aimed at collecting data through group interactions by exploring and sharing participants’ knowledge, experiences, and intuitions [26]. The interview guide providing the stages of the focus group, its aims and questions, is reported in Table 2.

A senior researcher (AP) led data collection with the support of a junior researcher (KT) in a calm environment facilitating interactions among participants without interruptions. The focus groups lasted on average one hour (from 45 to 130 min); they were audio-recorded and then transcribed *verbatim*. A total of 23 h of focus groups were audio recorded, verbatim transcribed and then analysed.

### Data analysis

Qualitative content analysis [30] was performed with the aim of understanding the manifest interventions and latent content of the data (e.g., the reasons why HCPs used each specific intervention) by three researchers (AP, TK, VB) and then supervised by other two researchers (MH, RW). In the coding process, researchers did not use the frameworks available in the field (e.g., Chang & Roberts [9]) to ensure the flexibility needed to examine the phenomenon in its natural state, as represented by the tacit knowledge of HCPs [24].

Specifically, the following analytical steps were undertaken (29,30): (a) the transcriptions were read and re-read carefully by researchers, and a first level of analysis was conducted by selecting those units of meaning; (b) then, each unit statement was coded in sub-themes initially independently and then reaching a consensus among researchers; thereafter, (c) the codes were categorised in themes and sub-themes, also in this case initially independently and then by common agreement; a full description of themes and subthemes was also provided through team discussion; and (d) the organization of the themes and sub-themes was then presented according to the

- chronological order of events as they emerged in the focus groups; and
- the progressive focus, whereby researchers choose to move either from describing the broad context of an event to particular cases (the resident).

The emerging sub-themes were supported by also indicating what focus group(s) reported each specific intervention (Nursing Home [NH] 4). Quotations from the transcripts, and the sources of the quotations, are reported in the findings to support theme and sub-theme credibility; also in this case, the focus group source was reported (e.g., NH 5).

In Table 3, strategies used to ensure rigour and trustworthiness have been summarised. These were implemented at different stages of the research process including: protocol development, data collection (AP, KT, MM, MH, RW), data analysis (AP, KT, VB) and member checking with participants of four focus groups (AP, MM, VB).

**Table 3:** Measures Undertaken to Ensure Study Rigour and Trustworthiness [25, 30]

- Aiming at evaluating whether the focus group interview guide was clear and understandable, one pilot focus group was performed by involving an NH not included in the final analysis; no changes of the interview questions were suggested; - Aiming at ensuring that researchers and focus group participants were not friends and/or colleagues in previous or current work experiences, before data collection the degree of friendship was assessed. No previous relationships emerged; - Aiming at preventing biases in interpretation, researchers bracketed their preconceptions regarding the phenomena under study by sharing their belief and knowledge; - Aiming at ensuring triangulation, three researchers conducted the entire process of data analysis in an independent fashion and then they were supervised by two researchers. At each step, they shared and agreed upon the findings; - Aiming at ensuring data dependability, the focus groups were numbered (e.g., focus groups Nursing Home 4 [NH 4]); thus, direct mention of the focus groups(s) who indicated each specific intervention was provided for each sub-theme; - Aiming at providing findings credibility, direct quotes were reported by indicating also the appropriate focus group source (e.g., NH 5). - Aiming at ensuring validity, sessions of member checking of the findings were performed by returning to participants of focus groups of four NHs. They have agreed with the final analysis, which was then considered valid.	

*NH* Nursing Home

## Results

**Fig. 1:**
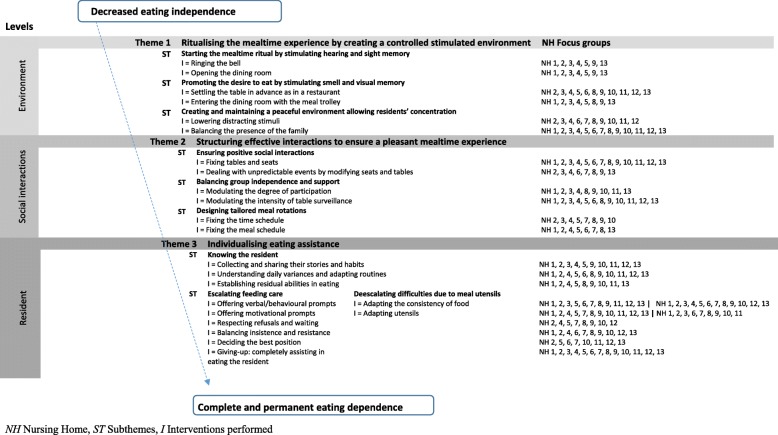
Interventions performed at the environmental, social, and individual resident levels aimed at maintaining eating independence in NH residents with dementia

The promotion and maintenance of eating independence for as long as possible is ensured by a set of interventions targeting three levels: (a) environmental, by ‘Ritualising the mealtime experience by creating a controlled stimulated environment’; (b) social, by ‘Structuring effective mealtime social interactions’; and (c) individual, by ‘Individualising easting assistance’ (Fig. 1).

### Theme 1. Ritualising the mealtime experience by creating a controlled stimulated environment

The first attempt by NH carers is to establish a routine to help older people recognise that mealtime is approaching and to prepare themselves to eat in an environment where different stimuli believed to increase their eating performance are controlled by staff.

#### Subtheme 1. Starting the mealtime ritual

In some NHs, healthcare professionals announce the start of mealtime by

“*…ringing a bell and then opening the dining room…*” (NH 2)

This ritual is performed three times a day, at breakfast, at lunch and at dinner time. In the few minutes before this, older people capable of approaching the dining room independently or by wheelchair usually queue outside the room; those unable to understand that it is time to eat are assisted to realise what is going on by accompanying them to the table, and/or by explaining that they are in the dining room and that it is time to have a meal.

By ringing the bell, the carers’ intent is to stimulate hearing memory based on Italian culture where church bells used to ring three times a day in the proximity of mealtimes. Opening the dining room as a space where the meals are offered is instead aimed at stimulating residents’ sight memory. These two interventions have been reported to function interdependently.

#### Subtheme 2. Promoting the desire to eat

Seeing a home-like dining room already equipped has been reported as an essential intervention promoting residents’ desire to eat. According to the experience of the interviewed HCPs, residents are not involved in the process of table and/or meal preparation because

“...*they prefer to be served*...” (NH 10)

Moreover, some older people do not like to eat meals prepared by other residents or served by other residents due to hygiene concerns. Additionally, given that roles have to be distinguished between those receiving and delivering care, the role played by residents is essentially to receive care.

Sight memory is stimulated again by entering an equipped dining room; the meal trolley is also taken into the room when all residents are already settled at their tables.

“*…entering with the trolley inside the hall, … it is better than the tray because they can choose what to eat…*” (NH 3)

Moreover, the smell of food can further stimulate their appetite, reinforcing their readiness for mealtime.

#### Subtheme 3. Creating and maintaining a peaceful environment

After the initial heavy stimulation, staff lower the stimuli to facilitate residents’ concentration on feeding. A calm environment is provided with different interventions

“*…we try to speak in a low voice, and also to move around the room slowly…*” (NH 11)

The most effective environment is one where only the light noises of dishes used by residents and conversation emerge; such a calm environment is also expected by residents. The television is switched off as well as music, as per request of the residents, and the television is switched back on at the end of lunch to signify that the mealtime ritual has ended.

Family members are allowed at mealtimes only when their presence is effective for both the individual resident and the entire group. Ineffective presence has been described when: (a) the family member is not compliant with staff recommendations (e.g., by helping with eating when not necessary, thus developing excessive dependence) or his/her behaviour is not in line with what is expected in the dining room (e.g., speaking softly and not disturbing other residents); (b) when the family member’s presence triggers the request of the same eating support by other residents; (c) generates residents’ agitation that occurs when they are in a hurry, thus stressing the residents during mealtimes; or (d) when the family member has not attended specific trainings. Instead, volunteers are considered extensions of the staff and allowed to be present during meal service only after appropriate training.

### Theme 2. Structuring effective social interactions ensuring mealtime as a pleasant social experience

The second set of interventions are aimed at creating pleasant social interactions among residents sitting at the same table during mealtimes by regulating three possible factors that can affect their eating performance: the relationship among residents; their degree of independence; and the meal rotations.

#### Subtheme 1. Ensuring positive social interactions

Personal relationships among residents, their preferences, sympathies, and friendships, as well as tensions or incompatibilities, are all continuously scrutinised by the staff, and according to their observations, they decide the table and seat of each resident. This decision is made collegially by the staff and tends to be permanent, thus allowing the residents to nurture positive interactions and to expect the meal experience to be substantially positive:

“*…we try to not introduce changes in the tables and in the seating arrangements unless absolutely necessary*...” (NH 9)

Residents with different levels of eating dependence who exhibit no disturbing behaviour are seated at the same table in front of each other to help those with poor performance to eat independently by mirroring movements observed in others. Differently, those residents who may display disturbing behaviours (e.g., spilling food out of their mouth) are seated at tables in the company of residents who are regarded by staff to be nonplussed by these manifestations (e.g., due to their cognitive decline). Furthermore, residents who prefer to eat alone have their wishes fully respected and are seated at a single table.

Tables and seats are modified only when unpredictable events occur, such as an

“*…episode of agitation or when behaviour generates tension, disgust, or discomfort among residents seated around the table...*” (NH 6)

Each change in table and chair arrangements is planned in advance and carefully conducted, because modifications in routines can trigger agitation that can further decrease eating performance both at the individual and group level.

The preferred meal table hosts at least four residents; large tables (with six or more seats) have been tested in some NHs with negative outcomes due to the complexity of the relationship of the residents who are forced to stay together.

#### Subtheme 2. Balancing group independence and support

Each table is approached by the meal trolley, and each resident is required to suggest preferences according to the options available on the menu, which is posted on the dining room door and also verbally reviewed by the carers at the moment of the resident’s involvement in the meal decision. In several NHs, menu preferences were used to be collected the day before or early in the morning; however, tensions appeared at meal service when residents forgot these preferences and requested other menu items, suggested involving them in expressing preferences at the time of the meal. Residents also influence each other; thus, a meal preferred by one resident is often preferred by others sitting at the same table. Therefore,

“*when asking the preferences of the first resident, healthcare workers consider if these options may be suitable for the others*...” (NH 4)

Moreover, some residents appear stressed when having several options offered: thus, at the moment of the request, healthcare staff tend to reduce the meal options by offering only a few.

During mealtime, staff is continuously present in the dining room, checking table by table, ensuring an unobtrusive supervision. They never leave the dining room until the end of the meal, and the intensity of their surveillance and support is delivered on the basis of the degree of dependence in eating.

#### Subtheme 3. Designing tailored meal rotations

The time when each resident is served and in what turn—before or after other residents—is not arbitrary. First, staff are used to balance the time required by each resident to complete the meal and their need to receive appropriate support: for example,

“*residents who require more time normally start lunch around half an hour earlier, thus allowing for individual support*...” (NH 10)

In other cases, other residents are served later because of their behaviour (e.g., a tendency to finish their meal earlier than others).

Among the group of residents sitting around a table, meal service order is also not causal: those who frequently refuse a meal because it appears different to the ones offered to those sitting near are served at the end whereas those who are impatient are served before.

Finally, in order to establish a routine, a meal schedule is also fixed: for example,

*“every Friday fish is served, and every 15 days pizza is the menu item*…*”* (NH 13)

This consistency creates expectations and the desire to eat.

### Theme 3. Individualising eating assistance

At the resident level, staff tailor interventions based on the preferences and habits of each resident, their daily variances, and by considering their actual feeding performance. These are considered baselines for the following interventions that involve escalating the intensity of eating assistance and decreasing difficulties generated by meals and cutlery and dishes.

#### Subtheme 1. Knowing the resident

At admission, staff collect meal preferences for residents as well as their personal stories and habits, interviewing family caregivers when the resident is not capable of answering. Establishing the actual degree of eating dependence aimed at maximising residual abilities is not easy: participants ensure a correct body position, given that from their experience, disability can also be determined by posture. Thus, data are shared with staff members and used to tailor daily care interventions: preferred meals are strictly respected, as well as residents’ habits (e.g., cooking) to form a basis of motivational prompts to stimulate eating. However, residents can have daily variances in their preferences as well as in their eating abilities for different reasons: for example,

“*…clinical instability…*” (NH 11)

“*constipation, and agitation*...” (NH 12)

thus triggering the need to immediately revise the individualised care based on emerging needs. These variances become clear in the morning when residents wake up and accompany them throughout the day.

#### Subtheme 2. Escalating eating assistance

Healthcare staff reported increasing assistance in eating by starting from prompts and then implementing different interventions until the decision to abandon all attempts when complete and permanent eating dependence appears. When self-feeding abilities become compromised, verbal, behavioural, and motivational prompts are offered. Although verbal and behavioural prompts are intended to simply stimulate the resident to recall movements and necessary sequences to eat independently, some prompts have been reported also to encourage a willingness to eat

“*…we promise sweets at the end of lunch…*” (NH 8)

Refusal to eat is respected: when it occurs, healthcare staff have reported waiting a few minutes to help other residents and then re-start the escalation of interventions by offering again verbal prompts. This strategy has emerged as effective according to the experience of participants; however, when the resident continues to refuse, staff is called to decide the degree of insistence by offering verbal and behavioural prompts. This is the point when the staff is called to decide to insist with prompts and with physical assistance (e.g., hand-to-hand) or to give up by suggesting enteral nutrition. From the perspective of time, staff may feel pressured to provide meals to the resident by physically assisting them as more efficient; however, when the NH is focused on preserving residual eating abilities, staff is used to avoid or postpone the complete assistance of the resident as long as possible.

In the later stages, a group decision is undertaken: this occurs when HCPs diagnose complete and permanent dependence in eating, which reflects a ‘point of no return’. Then, they evaluate the best position of eating assistance (on the right or left or face-to-face), which is ensured across all meals by the entire staff.

#### Subtheme 3. Deescalating difficulties generated by meal utensils

With increased dependence in feeding, participants have reported decreasing the complexity generated by food and cutlery:

“*…some food may be difficult to chew or digest, and some cutlery may be too difficult for residents to use*...” (NH 5)

Staff respond by offering foods with a softer consistency such as semi-liquid foods, or by providing residents with more simple utensils or methods of eating, especially spoons or offering finger foods. These decisions are immediately implemented when difficulties in eating start to appear; once these methods are decided upon, they become part of a routine for the given resident mainly due to the risk of adverse events such as aspiration.

## Discussion

HCPs adopt a set of interventions targeting three different levels such as the environmental, the social, and the individual levels. Recently, interventions have been categorized into environmental (changes in routine, context, and ambience) and behavioural (education or training of people with dementia or their caregivers) [14, 22], suggesting that in daily practice more levels are targeted. Moreover, these have been reported to be offered as a continuum and integrated with each other, thus reflecting a complex intervention framework [31] where different components work interdependently.

At the environmental level, NH carers are used to stimulating sight, hearing, and smell memory aimed at preparing residents through rituals by enacting a mechanism that seems to trigger a conditioned reflex [32, 33], trying to compensating some pathophysiological changes occurring due to dementia (e.g., visual and smell changes [7]). The ritualization of these interventions anchored in previous cultural patterns (e.g., ringing of the church bells near mealtimes) seems to be mixed with specialized interventions, such as tables already equipped by the staff as in a restaurant. The latter have been reported as an effective intervention despite studies reporting that involving residents in the process can increase their independence in feeding [34]. At the end of this intensive stimulus phase, in all NHs, healthcare professionals try lowering distracting stimuli to increase residents’ concentration on eating. The peaceful environment is created in light of the requests of residents where music is also avoided, suggesting something in contrast to findings of previous studies (e.g., Ho et al. [35]). Moreover, a peaceful environment is also ensured by the adoption of appropriate behaviour by the staff, which is also imposed upon family caregivers.

Although appropriate training of family members has already been highlighted [14] our participants underlined that they are involved in the process only when their behaviour is in line with what is expected and when their presence is positive for other residents as well, not just for their family member. Differently, permanent volunteers are considered part of the staff given their training and consistent behaviour as already documented [36].

At the social interaction levels, HCPs try to ensure a positive meal experience, as already suggested by previous studies in the field [3, 13]. However, the effective combination of residents at the same table is complex and based on different evaluations both of their positive and negative impact. Specifically, the staff considers: (a) the relationships among residents; (b) the reciprocal influence on eating abilities on the basis of a mechanism that seems to be based upon the mirror neurons theory [37]; and (c) the adverse behaviour that can occur on a daily basis (spilling food) or occasionally (aggressive manifestations). According to these complex elements, the size of the table is suggested to be effective when allowing four residents, different from the findings of Charras and Frémontier [38] who suggested from eight to 10 places.

Meal rotations have also been ritualised on a weekly basis; moreover, the order of meal serving has been reported as fixed. Decentralised food service at the table level (e.g., Bunn et al. [13]), as well as involving the residents in food choice [39] have already been documented in their effectiveness on feeding performance. However, preventing tensions among residents sitting at the same table by establishing an effective order of food choice and meal service both suggest that behind individualised care, feeding care interventions are also tailored at the group level. Moreover, the non-intrusive surveillance and supervision at the group level suggests that NH healthcare professionals are constantly focused on residents’ needs during the entire meal duration, differently from what has been documented at the hospital level [40].

At the individual level, three main interventions have emerged. The first is aimed at establishing preferences, habits, needs, and diagnosing the actual degree of eating independence. No diagnostic instruments (e.g., The Edinburgh Feeding Evaluation in Dementia Scale [20]) have been reported, possibly due to the long experience of participants and an in-depth knowledge of each resident. Given that eating independence is affected by daily variations, which need to be further investigated, the following interventions are decided on a daily basis by starting with verbal prompts.

The escalation of interventions has never been documented before, suggesting that care is progressively intensive and reaches a maximum degree of intensity when it is provided first through hand-over-hand help, and then completely supporting in eating the resident at each meal. Within the same process, the staff de-escalate the complexity of meal processes by modifying food and utensils. Previously Abdelhamid et al. [12] have already documented in their systematic review food modification as part of multi-component interventions.

Also the verbal and the behavioural prompts have been documented in the available literature [2, 3, 9, 12–14, 16–22]. Motivational prompts are offered to stimulate a certain behaviour (e.g., self-feed) for a certain reward (e.g., sweets), which can be considered an operant conditioning [41]. Differently, the effectiveness of waiting when the resident refuses to eat, resisting the temptation to feed the resident, and re-starting after a while with verbal prompts, have never been documented to date. In this field, the role of workload pressures (e.g., feeding the resident without waiting due to time constraints), as well as the amount of time involved in waiting (e.g., a few minutes or more) have never been discussed before.

Above all, NH staff have reported implementing multiple strategies based on a deep knowledge of their residents and their interactions: this seems to confirm the need to ensure both the stability of the dyad (carer and resident) [42] but also the stability of the entire staff to maximise this knowledge and the consequent decisions. This contrasts the tendency of some NHs to recruit temporary or informal staff [43]. Moreover, in our focus groups, a mixed staff was involved and the majority of them were healthcare assistants, confirming that mealtime care is mainly in their scope of practice, whereas academically educated professionals (e.g., nurses, physiotherapist) who are also prepared to access evidence-based resources are minimally involved. Consequently, the process of ‘knowledge utilization and translation’ aimed at retrieving, critically evaluating, and transferring the so-called explicit knowledge [44] represented by scientific evidence can be slowed down. The limited exposure to the mealtime by academic educated HCPs can also prevent the transformation of practical queries into research questions.

### Study strengths and limitations

After more than three decades of research in the field, no gold standards have been established [12], suggesting the need to gain new insights from practitioners by highlighting interventions perceived to be effective and thus potentially evaluable throughout well-designed studies. Therefore, we have designed a large multi-centred qualitative study aimed at highlighting the tacit knowledge [24] enacted on a daily basis to maintain eating performance among NH residents with dementia.

However, in the focus group, we included three to seven HCPs, despite an adequate group size having been suggested to range from four to 12 participants [26]. Different strategies have been adopted to include several participants, such as informing the staff with regards to the aims of the study and its potential contribution to the knowledge development; moreover, in all NHs, the time devoted to the focus group participation was considered as a part of their shift work. However, in four NHs (n. 1, 3, 6, and 8) facilities were small and all HCPs available (*n* = 3) were involved and participated; differently, in one NH (n. 12) the majority of the staff were not eligible due to their short experience (< 6 months) as established by the inclusion criteria assuming that this time period is required to develop the tacit knowledge [24].

The interviews were conducted by focusing on the interventions performed in the dining room and excluding those performed in bedrooms thus providing data only regarding the interventions performed for those residents eating in the dining room.

Although we have provided the verbatim transcriptions at the end of each focus group to share the main findings with the next focus group, data saturation [45] was not evaluated, and data collection was ended when all NHs approached were involved. Furthermore, we have considered only effective interventions, while those unsuccessful as experienced by the HCPs were not explored suggesting that further studies in the field could be designed. In this line, what is the decision undertaken if the preferred sitting option of one resident is not suitable for the other residents around the table, has not emerged and requires future research.

## Conclusions

In NHs, the self-feeding decline is juxtaposed with complex interventions targeting three levels: environmental, social, and individual. These interventions are regulated on a daily basis due to the variances that can occur in eating performance and need to be further investigated.

At the environment level, HCPs create a wave of stimuli triggering the desire to eat followed immediately by a peaceful environment where residents are allowed to concentrate: the controlled ritual that is established is anchored in well-known factors and other aspects requiring further study, such as the desire of residents to not be involved in the process of meal preparation and delivery, as well as the negative consequences of family members at both the individual and group residential levels. At the social interaction level, mainly established among those residents sitting around a table, NH staff manipulate those antecedents acknowledged as ensuring a positive meal experience, demonstrating that behind individualised care, the interventions in NHs are also tailored at the group level. Also, in this case, several interventions are implemented on a daily basis given their perceived effectiveness, such as the size of the table and the positioning of each resident around the table; these need to be further investigated given that no relevant studies have been performed. At the individual level, preferences and habits of each resident are considered by staff as well as daily changes in eating performance. At this level the intensity of eating assistance is modulated for each resident and over time it tends to increase, while difficulties caused by meals and utensils are decreased. This process of escalating and de-escalating interventions, which are interdependent, as well as the effectiveness of some specific interventions (e.g., waiting in cases of resistance or refusal to eat) have also never been documented before, thus suggesting further lines of research.

## Abbreviations

ADLs: Activities of Daily Living
ED: Professional Educator
F: Female
I: Interventions performed
M: Male
NA: Nurse Assistant
NHs: Nursing Homes
PHYS: Physiotherapists
RN: Registered Nurse
SD: Standard Deviation
ST: Subthemes
